# Research Progress in the Development of Vaccines Against *Riemerella anatipestifer*

**DOI:** 10.3390/microorganisms13102312

**Published:** 2025-10-06

**Authors:** Junxvan Lan, Shaopeng Wu, Lu Zhao, Fakai Li, Dongyi Xing, Fan Li, Hui Tian, Xiaoxue Yang, Shuhong Sun, Miaoli Wang

**Affiliations:** 1 Shandong Provincial Key Laboratory of Zoonoses, College of Veterinary Medicine, Shandong Agricultural University, Tai’an 271000, China; lanjunxvan825@163.com (J.L.); 13064068525@163.com (H.T.); 2 Shandong Provincial Center for Animal Disease Control and Prevention, Shandong Provincial Center for Zoonoses Epidemiology Investigation and Surveillance, Shandong Provincial Key Laboratory of Zoonoses, Jinan 250100, China; wsp@sdau.edu.cn (S.W.); zl19931206@126.com (L.Z.); lifakai@shandong.cn (F.L.); 13611313817@163.com (D.X.); 18561896069@163.com (X.Y.); 3 College of Veterinary Medicine, Qingdao Agricultural University, Qingdao 266109, China; lf08031313@163.com

**Keywords:** *Riemerella anatipestifer*, vaccine, inactivated vaccines, attenuated vaccines, genetic engineering vaccines

## Abstract

*Riemerella anatipestifer* (*R. anatipestifer*, RA) is a globally distributed pathogen responsible for duck serositis, an acute multisystemic disease whose infection leads to substantial economic impacts in duck production. There is currently no specific therapeutic drug available for effective treatment. Importantly, the severity of the disease is closely associated with multiple environmental factors, including feeding conditions, management practices, weather fluctuations, and air quality parameters. Furthermore, the prevalence of various serotypes is a matter of concern, and the emergence of multi-drug-resistant mutants through continuous use of various antibiotics is a major challenge. Recently, it has been reported that RA infects domestic ducks, turkeys, geese, wild birds and chicken, which leads to its remarkable influence on the healthy development of waterfowl breeding industry and even poultry breeding industry. Given these challenges, vaccination is essential for disease control. Various vaccine types are currently available, including but not limited to live vaccines, inactivated vaccines, subunit vaccines and vector vaccines. This paper provides a comprehensive review of the development of vaccines for RA.

## 1. Introduction

*Riemerella anatipestifer* (*R. anatipestifer*, RA) is a Gram-negative bacterium of the family *Flavobacteriaceae*, characterized by the absence of flagella or spores [1]. It produces a capsule and is capable of infecting domestic birds such as ducks [2,3], geese [4], and turkeys [5], and a recent report shows that it can infect hens [6]. This bacterium is distributed widely and is currently a major pathogen in waterfowl breeding. However, it is difficult to eradicate from duck farms worldwide [7]. The disease manifests through a range of symptoms, including fibrinous pericarditis, perihepatitis, airsacculitis, caseous salpingitis and meningitis. Transmitted primarily through the respiratory tract, this disease causes high mortality, stunted growth, resulting in severe economic losses for duck farmers due to elevated condemnation, downgrading and salvage rates [8]. The infection is. The mortality rate can reach 75% in ducklings less than eight weeks old [9,10,11]. Bacterial serotyping relies on the polymorphic diversity of surface antigens, primarily capsular polysaccharide (CPS) and lipopolysaccharide (LPS). These antigens which exhibit significant structural heterogeneity among strains within a species, enable differentiation into distinct [12]. The key targets for this classification are the K antigen (a CPS component) and the O antigen (an LPS component), and 25 serotypes have been reported so far [13]. The geographical distribution of predominant RA serotypes varies significantly, and the lack of cross-protection between them complicates disease management. For instance, serotype 10 predominates in Vietnam (alongside serotypes 1, 6, 8, 20) [14]; serotype 1 is most prevalent in Australia (with serotypes 6, 8, 13, 20) [15]; while China reports a broader diversity, with serotypes 1, 2, and 10 being most common among at least 12 documented serotypes [16,17].

Furthermore, the bacterium has gradually developed a high level of antibiotic resistance on a global scale, making vaccination the only feasible means of disease prevention by inducing an effective protective immune response in the host [18]. To achieve the desired level of decontamination, ducks must be regularly inspected and vaccinated. Vaccination is currently the primary method of preventing the occurrence of RA. RA vaccines can be broadly categorized into three main groups: live attenuated vaccines, inactivated vaccines and genetically engineered vaccines. In the context of advancements in next-generation vaccine development, there is a need for further evaluation of emerging screening methods, identified antigens, and unidentified antigens. This will facilitate the development of new vaccines to more effectively prevent RA. This paper provides a comprehensive review of vaccine development and a theoretical basis for novel vaccines with enhanced efficacy against RA in affected regions.

## 2. Conventional Vaccines

### 2.1. Inactivated Vaccines

The inactivated vaccine is considered to be one of the most cost-effective options. The inactivated vaccine has been shown to induce cellular and humoral immunity against RA infection in ducklings, with significant increases in IFN-γ and interleukin-2 (IL-2) production [9]. However, it has been demonstrated that the inactivated vaccine does not provide complete protection against RA infection in ducklings [19], mainly due to the different serotypes prevalent worldwide. Moreover, there is little or no significant cross-protection among the serotypes [20,21,22,23,24]. Consequently, serotyping of RA from field outbreaks and recognition of new serotypes are imperative for the effective management of the disease. In a recent report, Liang et al. prepared a new bivalent inactivated vaccine (WZX-XT5) containing a propolis adjuvant [25]. The dosage of 10^9^ CFU was found to provide ideal protection. Notably, WZX-XT5 immunization has been observed to elicit elevated levels of RA-specific IgY, IFN-γ, IL-2 and IL-4 in serum while conferring over 90% protection against RA in ducks exposed to an ultra-high lethal dose [25]. An earlier study reported that ducks given two immunizations with a trivalent inactivated RA vaccine, including serotypes 1, 2, and 10 were protected against RA strains from any of the three serotypes [26].

### 2.2. Live Attenuated Vaccines

In recent years, multiple studies have developed promising RA live attenuated vaccine candidates for RA. For instance, the attenuated vaccine RA CH-1 Δ*fur* has been demonstrated to elicit higher IgY titers, with serum antibody levels persisting for at least 49 days after immunization. However, the vaccine demonstrated favorable safety and protection against serotype 1 but not serotypes 2, 7, 10 and 11 [9]. Notably, Kang et al. screened two vaccine candidate strains, D15-RDA-92 (serotype 1) and D14-RDA-8 (serotype 2), which exhibited ≤50% embryo mortality in vitro and conferred significant protection against virulent serotype 1/2 strains at 21 days post immunization, with safety evaluations confirming tolerance to 100-fold dose administration [22]. Additionally, the strain RA CH-1 Δ*B739_1343* protected 83.33% of ducks against a 100 LD_50_ challenge from wild-type RA CH-1, suggesting its potential as a vaccine candidate [1]. With regard to the mechanisms of virulence attenuation, Guo et al. constructed a Δ*sprT* mutant lacking the T9SS core protein gene, which exhibited a 42,000-fold reduction in virulence compared to wild-type RA YM [27]. Similarly, Yu et al. identified an LPS-deficient mutant strain RA Δ*604* (with inactivated M949_1360), which exhibited >200-fold virulence attenuation in duck models and an 87.5% protection rate against the R strain [28]. Further investigations by Wang et al. revealed that deletion of the *AS87_01735* gene significantly attenuated RA Yb2 virulence (mutant RA625), while Yb2 Δ*pncA* immunization effectively protected ducks from wild-type Yb2 challenges [29]. More importantly, the B739-2187 deletion mutant provided 100% protection against wild-type RA, highlighting its critical value in vaccine development [30]. In order to achieve cross-serotype protection, Zou et al. generated the RA M1 strain (*M949_1603* glycosyltransferase gene inactivated) via Tn4351 transposon mutation [31]. Remarkably, ducks immunized twice with inactivated RA M1 vaccine achieved 100% protection against challenges from WJ4 (serotype 1), Yb2 (serotype 2) and HXb2 (serotype 10) [31]. Moreover, the CH3 Δ*M949_1556* mutant (with inactivated *M949_1556*) demonstrated broad-spectrum protection against the same three serotypes [32]. Nevertheless, despite promising experimental outcomes, the practical application of attenuated live vaccines of RA is not widespread due to concerns regarding the potential reversion to virulence and the impact on the quality of the feeding environment. Therefore, future research should prioritize optimizing genetic stability and comprehensively evaluating ecological effects to facilitate translational implementation.

## 3. Genetic Engineering Vaccines

Genetic engineering vaccines, encompassing diverse formulations developed through recombinant DNA technologies, demonstrate distinct preparation methodologies and application profiles. These include subunit vaccines, vector-based vaccines, DNA vaccines, synthetic peptide vaccines, gene-deleted vaccines, and transgenic plant-derived vaccines. While offering advantages such as enhanced safety profiles and cost-effectiveness compared to conventional vaccines, the production of genetic engineering vaccines necessitates advanced technological infrastructure and specialized equipment.

### 3.1. Subunit Vaccines

Recent advances in this field have focused on the development of recombinant subunit vaccines targeting immunodominant antigens, expressed in optimized systems. As is known to all, the outer membrane protein OmpA, a major RA immunogen [33], has been widely explored, though its protective efficacy varies considerably across serotypes [19,34,35]. To address this limitation, the fusion of OmpA and duck IgY Fc has been shown to enhance macrophage phagocytosis, amplify humoral and cellular immunity and provide protection against RA. Importantly, adjuvanted formulations incorporating *Schisandra chinensis* polysaccharide (SCP) further boosted macrophage activation and immune stimulation [19]. In contrast, GroEL protein-based vaccines exhibited limited cross-serotype protection (50%, 37.5%, and 37.5% against serotypes 1, 2, and 10, respectively) [36]. Intriguingly, Xu et al. demonstrated differential efficacy between recombinant OmpA variants, with rOmpA1164, but not rOmpA1467, providing partial protective immunity [35]. A recent study found that the combination of OmpA and CpG oligodeoxynucleotide (ODN) may represent a viable strategy for the development of a RA subunit vaccine capable of conferring long-term protection [37]. The subunit vaccine, comprising recombinant OmpA antigen (serotype 2), was formulated with CpG ODN as an adjuvant, with the capacity to enhance both humoral and cellular immunity. However, it should be noted that there are still critical gaps in the research, as the survival rates of the post challenge were not quantified or shown in these reports [21]. Promisingly, vaccination with recombinant YaeT (an outer membrane protein) achieved an 80% survival rate in Cherry Valley ducks challenged with arthritis-inducing RA strain [7]. Furthermore, under iron-limited conditions identified two novel antigens (Riean_1750 and Riean_1752), whose combined immunization conferred 100% protection against RA infection [38]. Similarly, Zhai et al. validated three recombinant proteins with high protection indices against RAf63 (serotype 1) and RAf153 (serotype 2) via immunoprotein histochemistry, highlighting their cross-protective potential [39]. Moreover, Huang et al. identified 12 immunogenic proteins from RA strain Yb2, with AS87_RS06600 immunization showing reactivity against serotypes WJ4, Yb2, and HXb2 [40]. The employment of pan-genome analysis in conjunction with reverse vaccinology has facilitated the systematic identification of cross-protective RA antigens. This approach successfully identified three recombinant proteins exhibiting immunoreactivity against five major serotypes (1, 2, 6, 10, and 11) [41]. Furthermore, anti-RA IgY antibodies have been demonstrated to possess the potential to enhance passive immunity and responsiveness in ducklings, thus offering a complementary strategy to active vaccination [42].

### 3.2. Recombinant Vector Live Vaccines

To date, the development of vaccines for ducks has been limited in scope, yet it has demonstrated considerable potential in terms of versatility across various pathogens. The primary utilization of viral vectors, including the duck plague vaccine [43,44,45], the influenza virus vaccine, the Newcastle disease virus vaccines [46] and the adenovirus vaccine [47]. In addition, some bacterial vector vaccines have been developed, such as *Bacillus subtilis* [48,49] and *Lactococcus lactis* [50,51].

### 3.3. Gene Deletion Vaccines

Emerging strategies in RA vaccine development focus on rationally attenuated strains through targeted gene deletions, particularly targeting virulence-associated pathways.

#### 3.3.1. Capsule and Outer Membrane Protein Targets

As demonstrated in several studies, live attenuated bacterial strains with defective capsules have been shown to effectively protect against the challenges posed by virulent strains [52,53]. Building upon these findings, Wu et al. successfully constructed two gene deletion mutants Δ*3820* and Δ*3830* targeting GE296_RS03820 and GE296_RS03830, respectively [54]. They further found that the ability of Δ*3820* and Δ*3830* to cross the blood–brain barrier was significantly reduced. Importantly, animal challenge experiments established that immunization with these attenuated strains conferred significantly enhanced survival rates in vivo. Immunological analyses further revealed broad-spectrum cross-reactivity between serum from mutant-immunized ducklings and multiple RA serotypes (1, 2, 7, and 10) [54]. Notably, comparative virulence assessment in strain Th4 (serotype 2) demonstrated that the Δ*ompA* mutant displayed a 22-fold attenuation in LD_50_ relative to its parental strain [55].

#### 3.3.2. Iron Acquisition System Targets

The TonB-dependent outer membrane receptor (TbdR1) has been identified as a cross-immunogenic antigen across RA serotypes 1, 2, and 10, thus highlighting its potential as a broad-spectrum vaccine target [23]. Mechanistically, TonB systems play a critical role in iron acquisition, with studies confirming that knockout of either *tonB1* or *tonB2* substantially attenuates bacterial virulence in duck models, as evidenced by reduced colonization capacity and pathogenicity [56,57]. Notably, virulence attenuation mediated by TbdR1 has been quantitatively characterized in vivo. Comparative LD_50_ analyses revealed a 10^4^-fold attenuation for the Δ*B739_1343* strain (Δ*tonB1*) and a 10-fold attenuation for the Δ*B739_1208* strain (Δ*tonB2*), relative to their parental strain [1,58].

#### 3.3.3. Regulatory Gene Targets

Utilizing a duck model in vivo, it was determined that the pathogenicity of the Δ*phoP* and Δ*phoR* mutants was significantly reduced compared to that of the wild-type strains [59]. The virulence of the mutant strain YM Δ*phoP* was reduced by approximately 47,000 times compared with the wild-type strain YM, while immunization with this attenuated mutant conferred 100% protection against subsequent wild-type challenges [18]. Building on these attenuation strategies, Wang et al. developed a CRISPR-Cas9 coupled mutant (YM Δ*Cas9*) showing impaired hepatic, cardiac, and hematogenous colonization capacities [60]. Notably, intranasal administration of YM Δ*Cas9* prior to wild-type exposure achieved 80% protective efficacy in ducklings, demonstrating its potential as a mucosal vaccine candidate [60]. Immunization with inactivated RA1062 lacking the M949_RS01035 gene has been shown to provide cross-protective IgY against serotypes 1, 2 and 10 [61].

#### 3.3.4. LPS Synthesis Pathway Targets

The mutant RA2640, which lacks this gene, has been demonstrated to exhibit a blocked LPS synthesis, altered serum agglutination ability, and other phenotypes, resulting in a significant reduction in virulence by more than 100,000-fold [62]. From the standpoint of developing a gene deletion vaccine, the RA strain with LPS-related gene deficiency has the potential to serve as a vaccine candidate to provide specific immune protection for animals while retaining adequate immunogenicity [62].

### 3.4. DNA Vaccines

As next-generation genetic immunization platforms, DNA vaccines employ engineered plasmid vectors to precisely deliver and express genes encoding immunodominant protective antigens. Current development of RA-specific DNA vaccines remains predominantly centered on optimizing OmpA expression, while plasmid-based formulations expressing alternative protective antigenic determinants remain underexplored. Notably, the evolutionarily conserved OmpH protein, a structurally exposed conformational epitope abundant in Gram-negative pathogens, demonstrates superior immunogenic potential through its capacity to elicit robust humoral responses and induce serotype-independent protective immunity [63]. Pioneering work by Gong et al. established groundbreaking proof-of-concept through development of a modular fusion construct co-expressing OmpA and OmpH [64]. This bivalent DNA vaccine demonstrated significantly enhanced protective efficacy against *Pasteurella multocida* challenge compared to monovalent formulations, achieving relative survival improvement in immunized hosts [64]. These findings fundamentally advance our understanding that multivalent antigen presentation through rationally designed polycistronic vectors enables synergistic immunostimulation, thereby overcoming the intrinsic limitations of single-epitope genetic vaccines.

## 4. Immune Program and Immunologic Adjuvant

Although inactivated oil-adjuvanted vaccines remain the most cost-effective prophylactic intervention against RA in ducklings, their efficacy is intrinsically limited in neonates due to immunological immaturity, achieving only partial protection during early developmental stages. To circumvent this limitation, strategic supplementation with levamisole as an immunostimulant has been shown to augment T-lymphocyte proliferative responses, correlating with a 30% relative improvement in protective efficacy compared to adjuvant-only controls. In order to prepare an effective vaccine, Liang et al. conducted several groups of animal experiments to optimize the immune conditions, such as the RA vaccine strain, immune dose and adjuvant [25]. The adjuvants commonly employed in clinical practice include propolis, oil emulsion and aluminum hydroxide. The findings of this study demonstrated that the combination of propolis and oil emulsion adjuvants elicited a more robust immune response in comparison to aluminum hydroxide adjuvants, likely through synergistic activation of both Th1 and Th2 immune pathways [25]. Innovative prime-boost regimens developed by Wu et al. demonstrated superior immunogenicity over traditional single-modality approaches: Sequential administration of DNA vaccine (pcDNA3.1-ompA) followed by recombinant OmpH subunit vaccine elicited sustained antibody titers persisting for 16 weeks post-immunization, significantly elevated the percentage of the cytotoxic CD8^+^ T cell and higher expression levels of IFN-γ, IL-6 and IL-12 mRNAs [34]. Furthermore, sera from prime-boost regimens especially DNA-prime and protein-boost were able to recognize lysates of RA serotypes 1, 2 and 6, indicating that it can produce cross protective effects [34]. The adjuvant combination of biodegradable calcium phosphate nanoparticles and Gram-negative bacterial outer membrane vesicles (OMVs) represents a promising strategy to elicit effective immunity, offering a superior alternative to conventional aluminum adjuvants. The coupling of calcium phosphate nanoparticles with outer membrane vesicles of RA has been employed in the development of a vaccine for ducklings [8]. The serum IgG and secretory IgA levels of ducks inoculated with CAP-OMV nanoparticles were higher than those of other immune groups, and the survival rate was 100% in the challenge experiment. Cytokines have been shown to play an important role as adjuvants in poultry vaccines. A notable example is IL-2, which has demonstrated potent adjuvant efficacy for an inactivated vaccine against Tembusu virus disease [65]. Duck IL-7, when employed as a novel adjuvant, has been shown to enhance the humoral immune response to an inactivated duck Tembusu virus vaccine [66]. Furthermore, the employment of IL-6 in the construction of Recombination vaccines has been demonstrated to be efficacious in the control of *Pasteurella multocida* [67].

## 5. Prospects and Summary

The prevalence of RA in numerous countries has resulted in the occurrence of septicemia and infectious serositis in domestic ducks, thereby causing substantial economic losses to the duck industry. Furthermore, there have been reports of cross-species transmission to chickens in recent years [6,68]. The extensive use of antibiotics to treat and control the disease has been a major contributing factor to the emergence of antibiotic resistance in RA. These strains demonstrate a high level of drug resistance, while the presence of antibiotic residues in poultry food has been demonstrated to pose a threat to global public health and safety [69,70,71]. It is imperative to recognize the gravity of the situation, as RA has become a substantial threat to the global duck industry, primarily due to its high prevalence rate and the development of drug resistance to various antibiotics [16]. The lists of available conventional and genetic engineering vaccines against RA infection are given in Table 1 and Table 2. At present, vaccination is considered the most effective strategy for the control of RA. Owing to their stability, cost-effectiveness, and capacity to provoke a potent and sustained immune response, inactivated vaccines are a promising and potentially efficacious option. It has been demonstrated that inactivated vaccines offer significant advantages in terms of safety and cost-effectiveness, but they are unable to provide cross-protection. Consequently, it is necessary to formulate vaccines following the prevailing serotypes [8]. This highlights the crucial importance of continuous local epidemiological monitoring in guiding vaccine strain selection. We believe that a more dynamic and region-specific approach is needed to formulate inactivated vaccines to keep up with the constantly changing serotype prevalence and improve their actual efficacy in this field, and efforts should be made to develop multivalent vaccines as a strategy to enhance protective efficacy. Moreover, the safety of live bacterial vaccines must be given due consideration [9,72,73]. A limitation of live vaccines is the potential to inhibit growth, in addition to the potential barrier of obtaining licensing approval from certain regulatory agencies [74]. Additionally, attenuated live vaccines impose stringent cold-chain requirements for transportation, posing a major impediment to their widespread use in resource-limited regions. Promising strategies to mitigate these limitations include the development of novel lyophilization techniques or thermostable formulations, which would reduce reliance on cold chain infrastructure and enhance vaccine accessibility and applicability. For vaccinated ducks, nano-selenium acts as a beneficial dietary supplement by enhancing growth performance, immune status and cytokine production [75]. In the context of next-generation vaccine development, further evaluation of the screening methods and identified antigens from the Protein Modules & Combinatorial Peptide Library will be instrumental in the development of new vaccines that will more effectively prevent RA. The multi-epitope vaccine has the capacity to carry multiple epitopes of the target antigen, and its preparation method involves the recombination of a plurality of DNA sequence fragments encoding antigen epitopes in series into a vector. It has been established that different serotypes of the same bacteria possess multiple epitopes, which have the capacity to induce specific immune responses in both B and T cells, in addition to the production of neutralizing antibodies [76]. Multi-epitope vaccines have been developed against several major poultry pathogens, including Newcastle disease virus vaccine [77], avian influenza A (H7N9) virus vaccine [78] and Marek’s disease virus vaccines (Herpesvirus of Turkey vector vaccine) [79,80]. High-quality antigen delivery systems have been put into use one after another, such as live attenuated typhoid vaccine [81] and adenovirus [82]. In particular, injectable vaccinations have been demonstrated to be incapable of triggering mucosal immunity [83]. Given that mucosal surfaces such as the nasal passages, oral cavity, throat, gastrointestinal and urogenital tracts serve as the primary entry routes for bacterial infections, mucosal vaccination represents a promising strategy to elicit protective immune response [84,85]. In addition, newly developed delivery systems have recently demonstrated high efficiency. For example, metal–organic framework-based nanovaccine delivery systems and covalent organic framework-based therapeutic platforms illustrate the growing application of nanoscale materials in biological fields [86,87]. Vaccines that induce strong mucosal immunity can substantially reduce infection rates and decrease the morbidity and mortality linked to infectious diseases. Nevertheless, there remains a critical shortage of approved mucosal vaccine adjuvants that are both safe and effective. The benefits of mucosal vaccines are manifold, including ease of management, improved compliance and straightforward administration in large-scale vaccination programs, even in the absence of medical training [88,89]. In recent years, the rapid advancement of artificial intelligence (AI) and nanotechnology has significantly accelerated innovation in vaccine development [90,91,92,93]. AI, particularly machine learning and deep learning algorithms, enables the rapid identification of immunogenic antigens and epitopes through the analysis of vast genomic, proteomic, and immunological datasets. Nanotechnology powerfully complements these advances by offering engineered nanoparticles (NPs)—such as liposomes, polymeric NPs, and biomimetic systems—that improve antigen delivery, stability, and immune activation. Further innovations, including virus-like particles and immune-stimulating complexes, enhance vaccine safety and efficacy by mimicking natural infection mechanisms to elicit robust and targeted immune responses. The integration of AI and nanotechnology unlocks extraordinary potential for developing personalized immunization strategies. The above new thoughts on RA vaccine will help to inspire basic and applied research in related fields and provide more candidate schemes for developing the next generation of safe and efficient RA vaccine.

## Figures and Tables

**Table 1:** The list of available conventional vaccines against *Riemerella anatipestifer* infection.

Types of Vaccines	Representative Vaccines	Key Features	Challenges/Limitations
Inactivated vaccine	Mono/Multivalent Vaccines (WZX-XT5 [25], trivalent inactivated RA vaccine (serotypes 1, 2, and 10) [26])	Cost-effective; Use of adjuvants	Poor cross-protection between different serotypes Requires customization based on local prevalent serotypes Does not provide complete protection
Live-attenuated vaccines	Various gene deletion strains (e.g., RA CH-1 Δ*fur* [9], D15-RDA-92 and D14-RDA-8 [22], RA CH-1 Δ*B739_1343* [1], RA YM Δ*sprT* [27], RA Δ*604* [28], Yb2 Δ*pncA* [29], RA M1 [31], CH3 ΔM949_1556) [32]	Attenuated by deleting virulence-associated genes	Risk of reversion to virulence Potential impact on feeding environment quality Not widely used in practice Requires optimization of genetic stability and evaluation of ecological effects

**Table 2:** The list of available genetic engineering vaccines against *Riemerella anatipestifer* infection.

Types of Vaccines	Key Antigens/Mechanisms/Vectors	Advantages	Challenges
Subunit vaccines	Main Antigens: *OmpA* [33], *GroEL* [36], *YaeT* [7], *Riean_1750/1752* [38], etc. Strategies: Fusion proteins (*OmpA*-IgY Fc), combination with adjuvants (SCP, CpG ODN) [19,37]	High safety profile Designed for specific antigens	Limited cross-protection with single antigens Need to identify more effective broad-spectrum antigens
Recombinant live vector vaccines	Viral Vectors: Duck plague virus [43,44,45], Influenza virus, Newcastle disease virus [46], Adenovirus [47] Bacterial Vectors: *Bacillus subtilis* [48,49], *Lactococcus lactis* [50,51]	Prevent multiple diseases simultaneously Induce stronger immune responses	Limited development for waterfowl
Gene deletion vaccines	Targets: Capsule/OMPs: e.g., Δ*ompA* [55] Iron acquisition systems: e.g., Δ*tonB1* [23] Regulatory genes: e.g., Δ*phoP* [59] LPS synthesis pathways: e.g., *RA2640* [62]	Rationally designed, clear virulence attenuation Retains immunogenicity	Need to balance attenuation degree and immunogenicity Require safety evaluation
DNA vaccines	Main Antigens: *OmpA*, *OmpH* [63] Strategy: Constructing multivalent vaccines	Precise design Relatively simple production	Current development is mainly focused on OmpA, and insufficient exploration of DNA vaccines for other protective antigens

## Data Availability

The original contributions presented in the study are included in the article; further inquiries can be directed to the corresponding authors.
